# Macrophages dynamically integrate neuro-immune-microbial network signals: their role in gastrointestinal motility disorders and targeting potential

**DOI:** 10.3389/fimmu.2026.1814676

**Published:** 2026-06-03

**Authors:** Weiqing Yang, Sunan Yong, Qiang Zhang, Wanjia Zhang, Qian Zhou, Xiaoqin Tan, Yin Xu

**Affiliations:** The First Hospital of Hunan University of Chinese Medicine, Changsha, Hunan, China

**Keywords:** enteric nervous system, gastrointestinal motility disorders, gut microbiota, macrophages, neuro-immune communication, therapeutic target

## Abstract

Gastrointestinal motility disorders are pathologically characterized by dysregulation of the tripartite network comprising the intestinal immune system, the enteric nervous system, and the gut microbiota. Targeted medicines that target important nodes in this network are desperately needed, and macrophages—tissue-resident immune cells with a high degree of plasticity—are showing promise as therapeutic targets. Through functional polarization, macrophages serve a key role in controlling intestinal inflammation and motility by integrating various signals from immune cells, pathogens, and neurons. According to new research, macrophages have two roles in gastrointestinal motility disorders: on the one hand, abnormal polarization can lead to immunological dysregulation and neuronal damage, which exacerbates motility dysfunction; on the other hand, certain subsets of macrophages aid in tissue repair and homeostasis. A thorough study that methodically outlines the pathogenic processes and therapeutic possibilities of macrophages in gastrointestinal motility disorders is still absent, despite recent advancements. In addition to evaluating new treatment approaches that target macrophage polarization, phagocytic function, and neuro-immune interaction, this review summarizes the unique mechanistic functions of macrophages in situations such postoperative ileus, constipation, and disorders of gut-brain interactions. In addition to offering theoretical insights and fresh approaches for precision intervention in gastrointestinal motility disorders, our goals are to expand on the present knowledge of the gut neuro-immune–microbiota regulating network.

## 1 Introduction

Gastrointestinal physiology is underpinned by a sophisticated anatomical structure and complex cellular connection systems (1). From an organizational standpoint, the gastrointestinal wall consists, from innermost to outermost layers, of the epithelium, lamina propria, muscularis mucosae, submucosa, muscular layer, and serosa, and is richly innervated by peripheral neurons. Based on their location, these neurons are primarily classified as those of the myenteric or submucosal plexus (2). They communicate via interneurons, motor neurons, and enteric glial cells to establish the core reflex units of the enteric nervous system (ENS), thereby coordinating essential physiological functions such as peristalsis and secretion.

Moreover, both the immune and neurological systems in the gastrointestinal tract have evolved the capacity to detect and swiftly react to alterations in the intestinal microenvironment. Nerve endings in the mucosa are frequently in close proximity to immune cells like macrophages, allowing for continual regulation and modulation by nutritional and microbial signals in the gut (3). The gut microbiota is now recognized as a crucial regulator of enteric nervous system development and function; commensal bacteria can elicit appropriate transcriptional and functional responses in neurons, thus preserving normal neuro-immune communication and intestinal homeostasis (4).

Nonetheless, disruption of this intricately coordinated interaction network can result in diseases primarily defined by gastrointestinal motility abnormalities, including disorders of gut -brain interactions, postop ileus, slow transit constipation, and gastroparesis. Network dysregulation is a hallmark of such conditions. Macrophages, leveraging their phenotypic plasticity and tissue-resident capacities, dynamically assimilate signals from neurological, immunological, and microbial origins. This review will examine the bridging and integrative roles of macrophages in the neuro-immune-microbial triad, systematically clarify their fundamental pathophysiological functions in gastrointestinal motility disorders, and investigate the potential implications of targeting macrophages for future therapeutic approaches and a more profound understanding of the intricate regulatory network of the gastrointestinal system.

## 2 Origin, classification, and function of intestinal macrophages

The intestine harbors the highest concentration of immune cells in the human body, and macrophages, owing to their remarkable phenotypic plasticity, serve as primary signal integrators and functional hubs (5). From the perspectives of origin and renewal patterns, most lamina propria macrophages are continuously replenished by circulating monocytes, resulting in a relatively rapid turnover rate (6, 7). Conversely, macrophage populations residing in deeper tissues, such as the myenteric plexus, submucosal plexus, and perivascular regions, are primarily derived from embryonic precursors, possess the capacity for local self-maintenance, and exhibit exceptionally prolonged lifespans (8).

Lamina propria macrophages (LpM) are pivotal for immunological surveillance (9). These cells are large and highly migratory, commonly express the CX3CR1 receptor, and extend transepithelial dendrites into the lumen to sample antigens (10). They are also responsible for clearing apoptotic cells (11), phagocytosing pathogens (12), and facilitating epithelial renewal through the production of mediators such as prostaglandin E2 (PGE2) (13). Their transcriptional profile favors the expression of pro-inflammatory genes; however, they also express receptors such as IL-10R to sustain immunological tolerance to benign antigens (14).

Macrophages deeply distributed within the muscular layer (muscularis macrophages, MMs) reside in close apposition to myenteric plexus (Auerbach’s plexus) neurons that regulate intestinal peristalsis (15). Morphologically, they appear as either bipolar cells aligned with nerve fibers or stellate cells surrounding ganglia. This population consists predominantly of long-lived, embryonically-derived macrophages that exhibit high expression of anti-inflammatory genes, including Arg1 and CD163, and display a transcriptional profile oriented toward tissue protection. Their primary function is to regulate ENS-driven gastrointestinal motility by producing bone morphogenetic protein 2 (BMP2) (16). Under stress conditions such as infection, they sense neural signals via β2AR to initiate neuroprotective programs involving Arg-1 and polyamines, thereby preventing neuronal loss, functionally resembling microglia in the central nervous system (17). Depletion of this population results in substantial neuronal loss and protracted intestinal transit.

**Figure 1:**
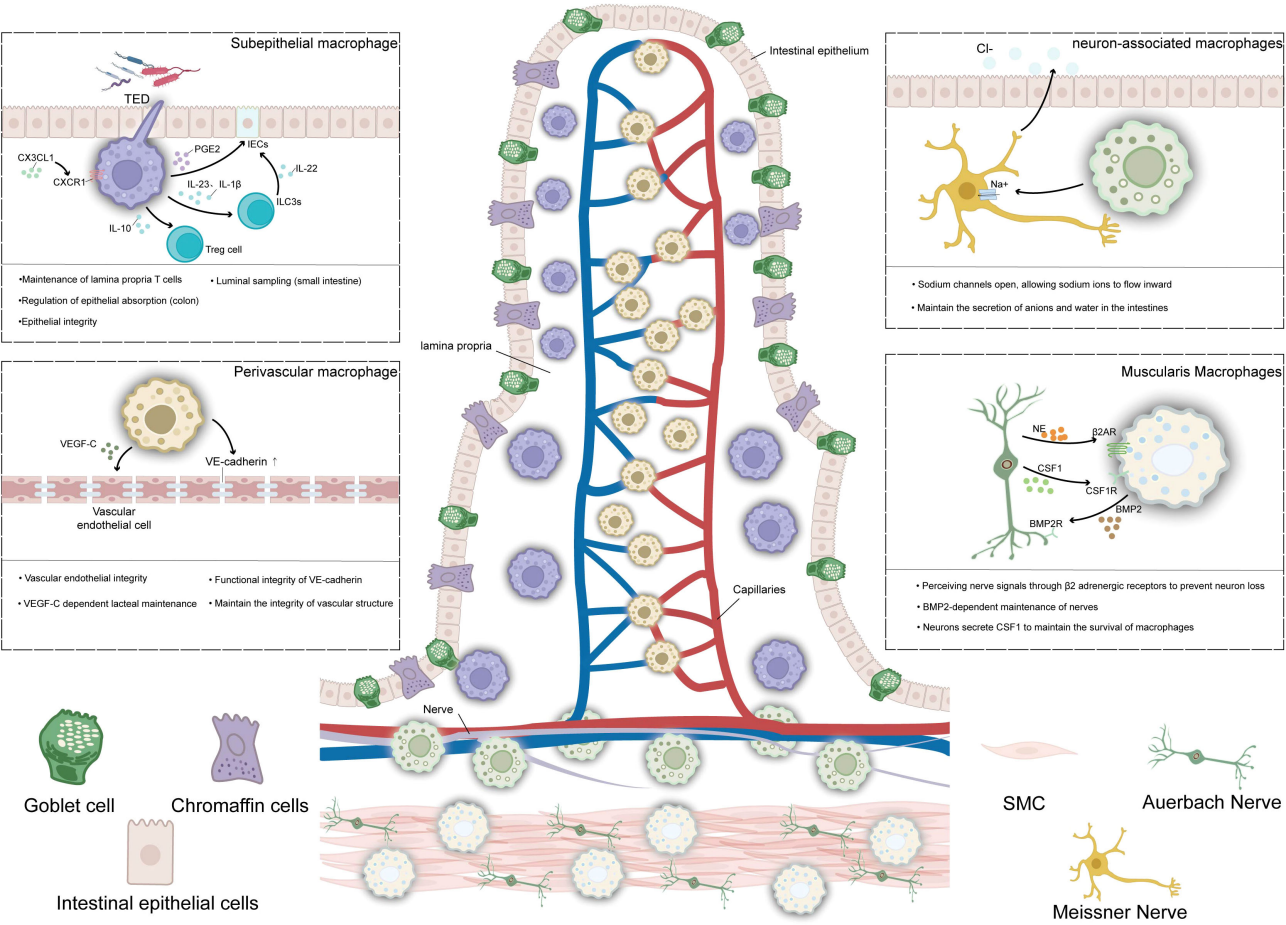
Functionally heterogeneous populations of intestinal macrophages. Intestinal macrophages are a very diverse group of defense cells. Because they are central signal integrators, their features and jobs are tightly controlled by the world in which they are found: Lamina propria macrophages (LpM) are at the front lines of immune surveillance and are in charge of removing antigens and renewing epithelial cells. Muscularis macrophages (MMs) are located close to enteric neurons and keep the gastrointestinal tract moving by secreting factors like BMP2 and protecting neurons. Other subsets are responsible for keeping the vascular integrity and supporting submucosal neurons. These groups have different origins, renewal patterns, and functional profiles. Together, they make up a well-coordinated cellular network that plays a key role in the interactions between neurons, the immune system, and microbes.

Additionally, numerous macrophage subsets reside throughout the intestine. Macrophages positioned near the villus capillary network (15) display a transcriptome profile characterized by elevated expression of genes associated with angiogenesis (18) and stromal remodeling (19). Functionally, they are directly involved in maintaining vascular structural integrity. They form tight associations with endothelial cells and support their maintenance by producing vascular endothelial growth factor C (VEGF-C) (20). Experimental targeted depletion of this population results in loss of the vascular endothelial junction protein vascular endothelial cadherin (VE-cadherin) and a marked increase in vascular permeability (15). These cells rely on continuous monocyte replenishment and require Nr4a1 and gut microbial signals for their development (21). Macrophages surrounding the submucosal plexus (Meissner’s plexus) primarily function to maintain submucosal neurons, thereby regulating intestinal anion and water secretion, which influences the lubrication and flow of luminal contents (15). Dysfunction of this cell type may indirectly decrease transit efficiency by altering the luminal environment. In conclusion, intestinal macrophages are not merely individual phagocytes; rather, they represent a highly specialized population of immune cells whose functions are intricately shaped by their specific tissue microenvironment (summarized in **Figure 1**).

## 3 Regulatory networks of macrophage phenotype switching

### 3.1 Phenotypic shaping of macrophages by signals from the enteric nervous system

The gastrointestinal tract and its associated nervous system meticulously govern tissue-resident macrophages through the secretion of diverse signaling molecules. During infection, activated sympathetic neurons release norepinephrine (NE), which signals through β2-adrenergic receptors (β2AR) highly expressed on muscularis macrophages, thereby promoting the expression of tissue-protective genes such as Arg-1 (16). *In vitro*, co-culture experiments demonstrate that enteric neural signals can induce macrophages to adopt a phenotype resembling that of muscularis-resident cells via a β2AR-dependent pathway (16).

Conversely, vagal anti-inflammatory signals stimulate cholinergic myenteric neurons to release acetylcholine (ACh) locally (22). Acetylcholine interacts with the α7 nicotinic acetylcholine receptor (α7nAChR) on macrophages (23), activating the Jak2/STAT3 pathway (24), which markedly inhibits the synthesis of pro-inflammatory cytokines including TNF-α and IL-6, while leaving IL-10 unaffected. Macrophage-specific deletion of α7nAChR completely abolishes the anti-inflammatory and pro-motility effects of vagal nerve stimulation or nicotinic receptor agonists in postoperative ileus.

Additionally, several neuropeptides released by intestinal neurons and endocrine cells influence macrophage activity. For instance, 5-hydroxytryptamine (5-HT) can activate the Wnt/β-catenin pathway through its receptor (13), facilitating PGE2 release to promote epithelial regeneration, calcitonin gene-related peptide (CGRP) encourages polarization toward an anti-inflammatory phenotype (25), neuropeptide Y (NPY) can enhance antigen presentation and pro-inflammatory cytokine synthesis (26).

Enteric glial cells serve a crucial intermediary function within this network. Activated glial cells can secrete C-C motif chemokine ligand 2 (CCL2) to attract monocytes and facilitate their differentiation into a reparative CD206^+^MHCII-high macrophage subpopulation via release of colony-stimulating factor 1 (CSF-1) (27). The anti-inflammatory properties of the vagus nerve may depend on a functional glial cell network for signal transmission (28).

**Figure 2:**
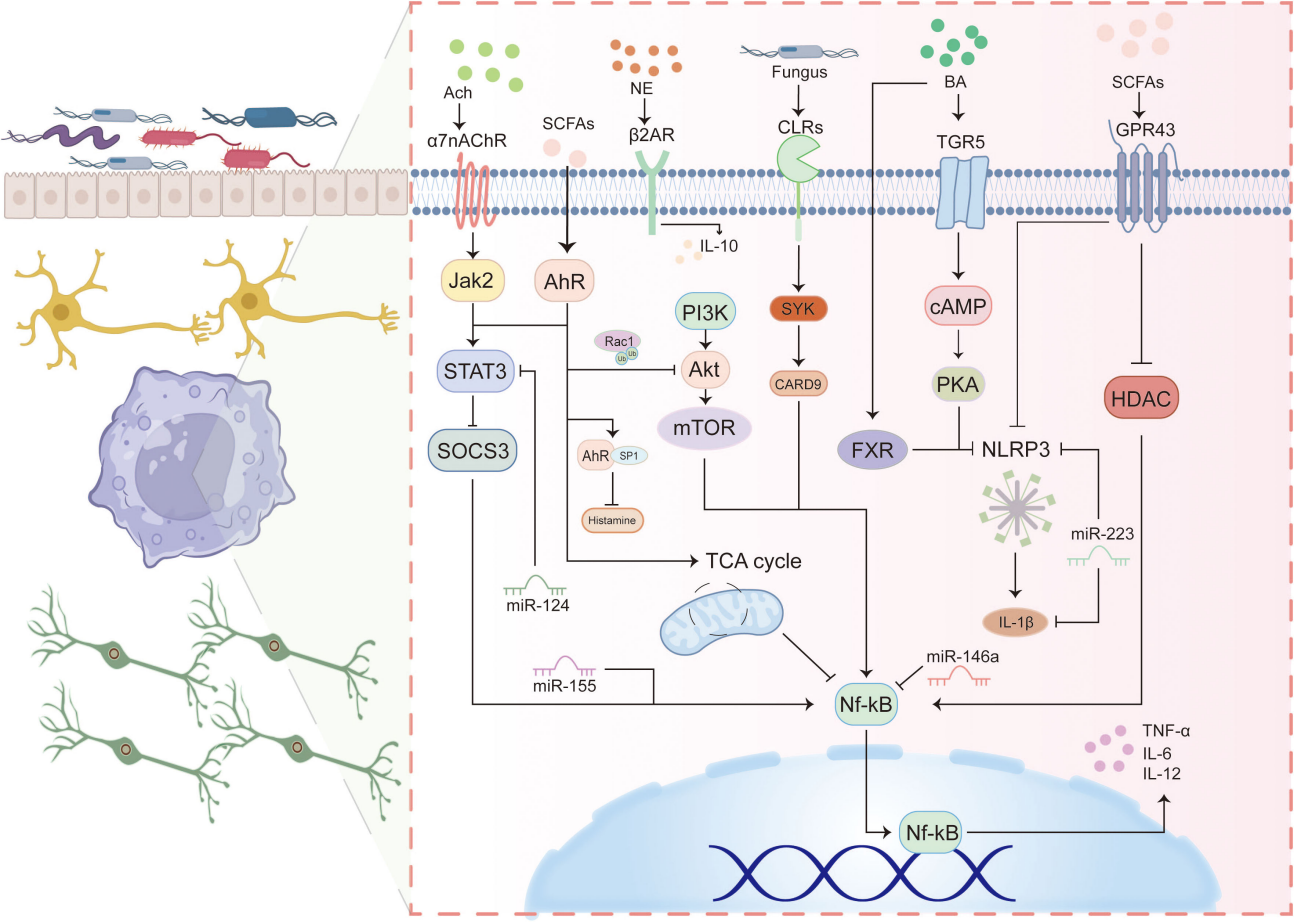
Signaling programming of intestinal macrophages. Macrophages play a key role in connecting the nervous system, immune system, and microbiota. Their phenotype and function are carefully controlled by multiple aspects of the intestinal microenvironment: (1) Nervous system signals, such as NE, ACh, and various neuropeptides, directly modulate cellular activation states through specific receptors including β2AR and α7nAChR. (2) Microbiota-derived metabolites, including SCFAs, indole derivatives, and secondary bile acids, influence epigenetic and metabolic programming via GPR43, AhR, and TGR5, promoting anti-inflammatory and reparative states. (3) Epigenetic regulation, mediated by miRNAs such as miR-146a, miR-155, and miR-223, fine-tunes the balance between pro- and anti-inflammatory responses by targeting key signaling molecules. Collectively, these regulatory networks illuminate the involvement of macrophages in gastrointestinal motility disorders and provide a framework for developing targeted therapies.

This neuro-immune regulatory network matures progressively after birth. Research indicates that in neonatal Ret knockout mice lacking an enteric nervous system, muscularis macrophages nonetheless infiltrate and mature effectively (29). Their initial CSF-1 is derived primarily from endothelial cells and interstitial cells of Cajal, and they are capable of mounting a typical immune response to LPS stimulation. In conclusion, mature intestinal macrophages can dynamically integrate information from neurons and glial cells and translate brain activity into immunological responses because they express a wide variety of neurotransmitter receptors. However, CSF-1 from endothelial cells and Cajal interstitial cells is necessary for the early formation of this network (summarized in **Figure 2**).

### 3.2 Phenotypic programming of macrophages by microbiota and metabolites

The gut microbiota is a critical determinant of the composition and function of intestinal macrophages (4). A healthy microbiome is essential for sustaining macrophage populations and the synthesis of molecules vital for gastrointestinal motility, including CSF1 and BMP2. The microbiota transforms dietary components into various bioactive metabolites, such as short-chain fatty acids (SCFAs) (30), secondary bile acids (31), and indole derivatives (32). These compounds are recognized by specific receptors on macrophages, including G protein-coupled receptor 43 (GPR43), Takeda G protein-coupled receptor 5 (TGR5), and aryl hydrocarbon receptor (AhR), thereby enabling direct modulation of macrophage function.

Short-chain fatty acids can function as histone deacetylase inhibitors, facilitating macrophage polarization toward an anti-inflammatory phenotype via epigenetic reprogramming (33). The underlying mechanisms (34) include inhibition of the NLRP3 inflammasome (35), downregulation of pro-inflammatory cytokine production (33), and metabolic reprogramming toward oxidative phosphorylation (36). Indole derivatives produced from tryptophan metabolism serve as endogenous ligands for AhR (37). Their activation promotes ubiquitination and degradation of Rac1, suppresses inflammatory signaling pathways including PI3K/Akt/mTOR and NF-κB, reduces synthesis of pro-inflammatory cytokines such as IL-6, and concurrently enhances IL-10 expression through STAT3 signaling. AhR activation also promotes fatty acid β-oxidation to support an anti-inflammatory state (38). Moreover, activated AhR forms a complex with the transcription factor SP1, thereby suppressing histamine synthesis and attenuating inflammatory responses (39). Secondary bile acids activate the membrane receptor TGR5 and the nuclear receptor FXR (40), leading to NLRP3 inflammasome disruption and inhibition of pro-inflammatory gene transcription (41).

Additionally, fungal components in the gastrointestinal tract can be detected by C-type lectin receptors on macrophages, triggering immune responses through the SYK/CARD9 pathway (42). Fungi such as Candida albicans can activate the NLRP3 inflammasome, facilitating IL-1β release and thereby exacerbating inflammation (43). Disease-oriented research underscores the significance of this network. In certain irritable bowel syndrome patients, expansion of CX3CR1^+^ macrophages and the associated Th17 pathway has been observed (44). In Candida colonization models, Th17 cell expansion is dependent on intestinal CX3CR1^+^cells, suggesting that dysfunction of the fungus-macrophage axis may contribute to disease pathogenesis (45).

The gut microbiota and its metabolites precisely regulate macrophages at various levels—developmental colonization, epigenetic programming, metabolic remodeling, and functional polarization—via a complex network of receptors and signaling pathways, forming a fundamental mechanism for sustaining intestinal immune homeostasis (summarized in **Figure 2**).

### 3.3 Precision regulation of macrophage phenotype by epigenetics

As key epigenetic regulators, miRNAs are crucial for enabling macrophage phenotypic plasticity, establishing adaptive memory, and orchestrating precise responses to complex signaling networks. Numerous miRNAs act as molecular switches in regulating the balance between inflammatory activation and resolution by targeting specific signaling molecules. For example, miR-146a negatively modulates the NF-κB signaling pathway by targeting IRAK1 and TRAF6, thereby restraining excessive inflammation and facilitating restoration of immune homeostasis (46). Conversely, miR-155 promotes macrophage activation and fosters inflammatory responses and pathogen clearance by downregulating SOCS1 and SHIP1 expression (47). Moreover, miR-223 specifically targets NLRP3 and IL-1β, thereby dampening inflammasome activity and preventing excessive inflammatory activation (48). miR-124 restricts macrophage activation by inhibiting STAT3 and CEBPα (49, 50).

One of the primary diagnostic symptoms of irritable bowel syndrome (IBS) is abdominal pain, which is primarily caused by visceral hypersensitivity. Notably, PI-IBS (often diarrhea-predominant) and IBS-D are distinct entities. In PI-IBS patients, concurrent increases in TNF-α and miR-155 levels in colonic tissues have been noted (51). Animal studies suggest that blocking TNF-α can ameliorate the visceral hypersensitivity induced by miR-155 overexpression. Furthermore, studies indicate that miR-199 can influence visceral pain by enhancing translation of the pain receptor transient receptor potential vanilloid 1 (TRPV1) (52). Epigenetic reprogramming of macrophages is also profoundly influenced by the local intestinal milieu, including microbial metabolites. Butyrate, by inhibiting histone deacetylase activity, can epigenetically stabilize the anti-inflammatory gene expression profile in macrophages (33).

In conclusion, the critical involvement of specific miRNAs in modulating macrophage function and the pathogenesis of gastrointestinal motility disorders suggests that intervention strategies targeting these miRNAs and their associated signaling pathways represent promising avenues for future development of novel therapies that can regulate macrophage function, enhance tissue repair, and restore intestinal homeostasis (summarized in **Figure 2**).

## 4 Functional output of macrophages as network integrators in gastrointestinal motility disorders

### 4.1 Regulators of visceral sensitivity

**Figure 3:**
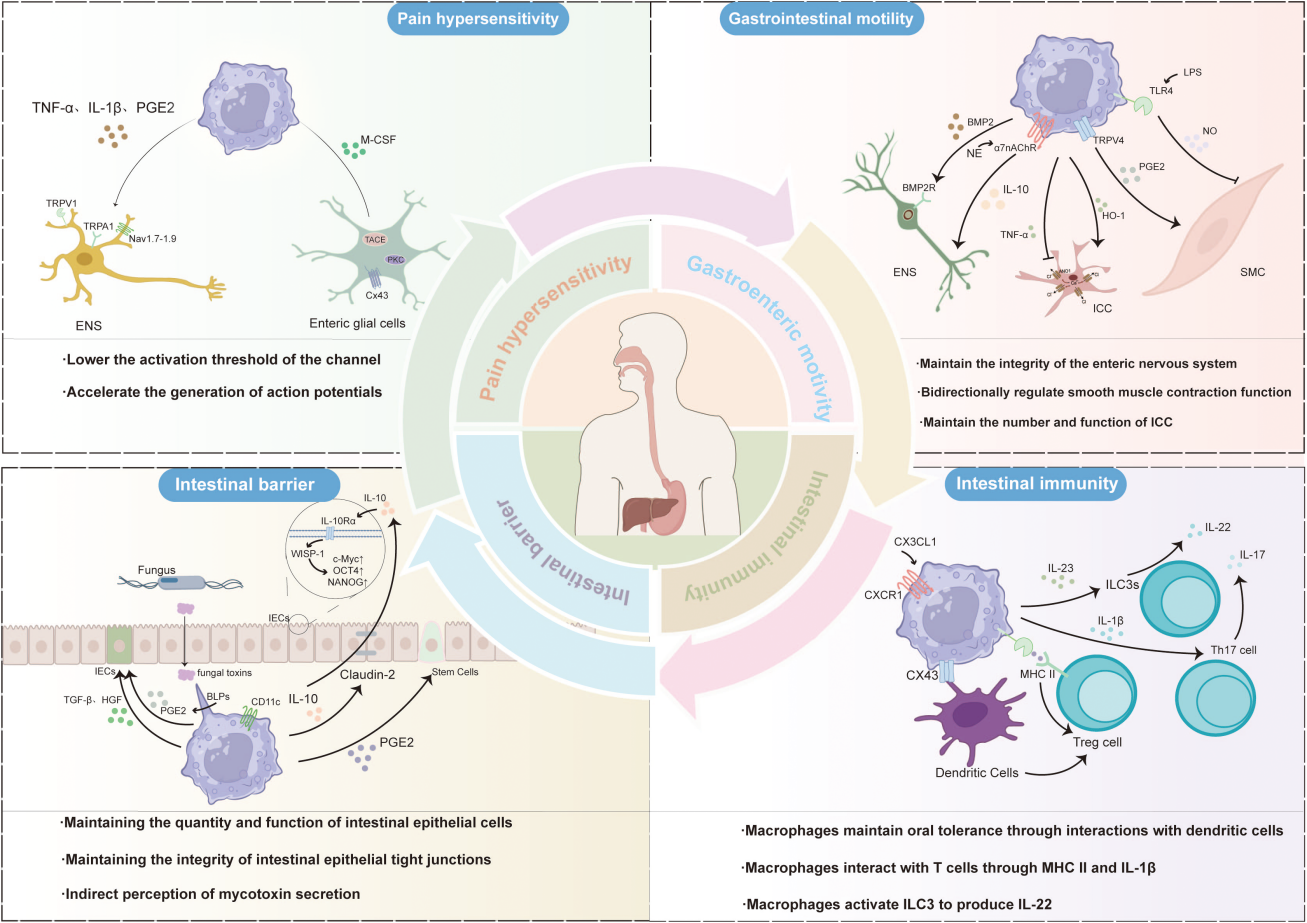
Functional output of intestinal macrophages. As central hubs of the neuro-immune-microbe tripartite network, intestinal macrophages regulate gut function through multiple distinct mechanisms. At the sensory level, they promote visceral hypersensitivity by releasing inflammatory mediators that sensitize nociceptors, either directly or indirectly via enteric glial cells. They fine-tune gastrointestinal motility through interactions with enteric neurons and by modulating smooth muscle contraction and interstitial cell of Cajal (ICC) function. For barrier maintenance, they protect against toxins and preserve epithelial integrity by releasing cytokines such as IL-6, IL-10, and PGE2, and by monitoring the microenvironment through basolateral extensions. To sustain immune homeostasis, they regulate the Treg/Th17 balance, communicate with ILC3s, and present antigens.

Visceral hypersensitivity is a primary pathogenic feature of disorders of gut-brain interaction (DGBI), characterized by heightened sensitivity of peripheral nociceptors. Recent studies reveal that this process is intricately linked to disruptions in the intestinal neuroimmune milieu, in which macrophages assume a pivotal role (summarized in **Figure 3**).

#### 4.1.1 Direct neural sensitization

During inflammation, macrophages recruited to the submucosa or muscle layer become activated and secrete pro-inflammatory mediators, including TNF-α, IL-1β, and prostaglandins (53). These mediators can directly engage specific receptors on nociceptors of primary afferent neurons in the gastrointestinal tract (54). By altering the activity and expression of membrane channel proteins such as TRPV1, transient receptor potential ankyrin 1 (TRPA1), and voltage-gated sodium channels Nav1.7–1.9, they substantially lower the neuronal excitation threshold and enhance sensitivity to mechanical distension and chemical stimuli, thereby directly inducing hyperalgesia. In later stages of inflammation or in response to specific signals, macrophages can transition to a pro-resolving phenotype, secreting anti-inflammatory mediators such as IL-10 and resolvins (55). These molecules can directly suppress nociceptor activity, thereby facilitating pain resolution (56). Consequently, persistent visceral hypersensitivity may reflect a dysregulated state characterized by pro-inflammatory macrophage predominance and compromised anti-inflammatory capacity.

#### 4.1.2 Indirect regulation via enteric glial cells

Research indicates that inflammation-activated intestinal glial cells can function as upstream regulators (57). They secrete M-CSF through their Cx43-PKC-TACE signaling axis. This factor can directly stimulate adjacent muscularis macrophages, ultimately leading to sensory neuron sensitization and the development of visceral hypersensitivity. This cascade illustrates how macrophages, as downstream effectors of glial cells, contribute to the emergence of visceral hypersensitivity.

In a model of post-infectious irritable bowel syndrome, levels of pro-inflammatory cytokines including IL-5, IL-6, IL-17, and TNF were significantly elevated compared to controls and correlated with clinical symptom severity (58). Additionally, notable immune cell infiltration within the intestinal mucosa and their proximity to sensory nerve fibers were observed, suggesting that immune cells such as macrophages may participate in the regulatory process. During the remission phase of a DNBS-induced intestinal inflammation model, a state of localized immune activation persists, evidenced by elevated numbers of mast cells and MHC-II^+^ macrophages (59). These cells are frequently situated adjacent to substance P^+^ nerve axons. This ongoing peripheral signaling subsequently activates glial cells at the spinal level, potentially perpetuating visceral hypersensitivity and intestinal dysfunction by influencing sensory integration and motor output.

In summary, intestinal macrophages, as key players in neuroimmune interactions, can directly sensitize nociceptors through release of inflammatory mediators and also modulate neuronal function via enteric glial cells.

### 4.2 Regulators of gastrointestinal motility

Muscularis macrophages reside primarily within the myenteric and deep muscular plexuses (60). Their anatomical positioning places them in close proximity to neuronal cell bodies and nerve fibers of the gut, frequently exhibiting a bipolar or stellate morphology aligned along nerves. This specialized spatial distribution establishes the anatomical foundation for monitoring neuronal activity and receiving neurosecretory signals, positioning them as a vital link between the enteric nervous system and the immune system. CSF-1 released by neurons is a crucial factor for the survival and maintenance of this population. In a feedback loop, muscularis macrophages release BMP2, which acts on enteric neurons to modulate the rhythm of intestinal peristalsis. This interaction is influenced by the gut microbiota, as commensal microorganisms can enhance BMP2 and CSF-1 expression. Moreover, these macrophages detect NE signals from sympathetic neurons through their expressed β2AR. This pathway enhances expression of tissue-protective genes such as Arg-1, thereby reducing neuronal damage and loss during infection or inflammation. This cell type also continuously clears apoptotic neurons, which is essential for maintaining stable neuronal numbers (61).

Additionally, muscularis macrophages can modulate gastrointestinal motility by directly affecting intestinal smooth muscle and interstitial cells of Cajal (ICC). Under physiological conditions, they detect mechanical or chemical stimuli via expressed TRPV4 channels, resulting in PGE2 production (62). PGE2 then stimulates intestinal contraction by engaging specific receptors on smooth muscle cells. However, upon inflammatory stimulation, these cells can be induced to express inducible nitric oxide synthase (iNOS) (63). Nitric oxide (NO) subsequently reduces smooth muscle contractility, leading to diminished intestinal motility. This indicates that muscularis macrophages exert dual regulatory effects on smooth muscle activity through distinct pathways. Moreover, pro-inflammatory M1-type macrophages can induce ICC cell death or reduce their activity through secretion of pro-inflammatory cytokines, thereby impairing their role in regulating gastrointestinal motility (64). Conversely, M2-type macrophages release IL-10, which promotes heme oxygenase-1 (HO-1) production and exerts a protective effect on ICC (65).

In response to enteric pathogens, muscularis macrophages exhibit remarkable functional plasticity (66). Certain infections can elicit a type 2 immune response, leading to eosinophil recruitment and prompting muscularis macrophages to adopt a persistent, neuroprotective state characterized by elevated Arg-1 expression. This functional adaptation can be sustained long-term, even after pathogen clearance, aiding in neuronal protection and maintenance of normal intestinal motility.

Impairment of muscularis macrophages is intricately linked to the development of numerous gastrointestinal motility disorders. Single-cell transcriptomic analysis of gastric muscularis from patients with diabetic gastroparesis reveals a marked decrease in macrophage subsets expressing tissue-protective genes, whereas receptors associated with monocyte infiltration and pro-inflammatory pathways are aberrantly activated (67). This indicates a shift whereby homeostatic, neuroprotective macrophage subpopulations are lost and replaced by monocyte-derived macrophages with pro-inflammatory capabilities. These cells ultimately release pro-inflammatory molecules such as TNF-α, damaging the ICC network (68)—the gastrointestinal pacemaker cells—and resulting in delayed gastric emptying. In post-infectious irritable bowel syndrome (69), pathogen infection may directly impair neurons through inflammasome pathways. In this context, muscularis macrophages are essential for neuroprotection via the β2AR/Arg-1 pathway, and their impairment leads to motility disturbances and sensory abnormalities (17). Contrary to common belief, in postoperative ileus—an acute injury model—surgical trauma primarily activates local muscularis macrophages rather than lamina propria macrophages, initiating early inflammation (70). Subsequently, monocyte-derived macrophages recruited via CCR2-mediated chemotaxis assume a reparative role by producing molecules such as Arg-1 and IL-10, clearing neutrophils, facilitating inflammation resolution, and restoring neuromuscular function. Absence of this reparative recruitment results in prolonged disease course.

In conclusion, muscularis macrophages continuously integrate diverse signals from enteric neurons, commensal microbiota, and the local immune microenvironment. Through this integration, they maintain the equilibrium of intestinal motility and neuroprotection under homeostatic conditions while activating adaptive protective mechanisms following infection or injury (summarized in **Figure 3**).

### 4.3 Guardians of the intestinal barrier

Intestinal macrophages contribute to epithelial integrity, tissue repair, and responses to environmental stress through diverse active mechanisms. Under physiological conditions, macrophages meticulously maintain the epithelial barrier by secreting multiple cytokines. Their production of IL-6 enhances expression of the intestinal epithelial tight junction protein Claudin-2 (71). This protein can form selective channels to modulate intestinal cation and water transport and participates in host defense. PGE2 released by macrophages, as a fundamental signal within the crypt stem cell niche, directly promotes survival, proliferation, and self-renewal of Lgr5^+^ intestinal stem cells, macrophage depletion results in reduced numbers of these stem cells (13). Furthermore, macrophage-derived IL-10 stabilizes tight junction protein complexes, reduces barrier permeability, and suppresses chronic inflammation that could compromise the barrier, thereby preventing translocation of pathogens and toxins (72). Epithelial cells detect macrophage-derived IL-10 signals through membrane receptors, leading to CREB activation via STAT3 phosphorylation, which enhances WISP1 secretion, increases expression of proliferation-related genes such as c-Myc, OCT4, and NANOG, and ultimately stimulates intestinal epithelial cell proliferation, renewal, and re-epithelialization (73).

Macrophage function is particularly critical during tissue injury and regeneration. By secreting growth factors such as HGF and TGF-β in a non-contact-dependent manner, they enhance epithelial cell migration and facilitate *in vitro* wound repair of the intestinal epithelium. Hepatocyte growth factor (HGF) (74) primarily stimulates rapid epithelial cell proliferation to promote wound closure, whereas transforming growth factor-beta (TGF-β) (75) boosts proliferation and induces cell differentiation, ensuring proper integration of newly formed epithelial cells into the epithelial structure. STAT6-mediated activation of Wnt signaling is also crucial for intestinal epithelial healing (76).

Moreover, to cope with the high microbial burden and complex environment of the intestinal lumen, a specific subset of macrophages with high CD11c expression does not directly contact luminal contents (44). Instead, they extend slender basolateral projections toward the basolateral aspect of the epithelium to “sample” fluid absorbed basolaterally, thereby indirectly monitoring the intraluminal environment. This process is particularly important for protection against threats such as fungal toxins. Upon detecting elevated toxin concentrations in the fluid, these basolateral projections enable macrophages to signal epithelial cells, through molecules such as PGE2, to downregulate activities like aquaporin expression, thereby reducing absorption of toxic fluid. This effectively prevents epithelial cell intoxication and apoptosis, preserving barrier integrity at its source.

In pathological settings, macrophage development and regeneration depend on CSF-1 receptor signaling. Blocking this pathway impairs epithelial healing capacity, underscoring its essential role in maintaining tissue homeostasis. In irritable bowel syndrome patients, the intestinal barrier frequently exhibits heightened permeability (77). Barrier disruption leads to antigen translocation, initiating immune responses and exacerbating low-grade inflammation. Under these conditions, pro-inflammatory macrophages are activated to address the threat (78), however, their sustained activation may amplify inflammation and compromise barrier integrity. Consequently, promoting macrophage polarization toward an anti-inflammatory, reparative phenotype is crucial for disrupting the inflammatory cycle, restoring the compromised barrier, and alleviating clinical symptoms (79).

In summary, through secretion of diverse cytokines and performance of distinct physical surveillance activities, intestinal macrophages collectively form a multifaceted, dynamic barrier defense and repair system. This system thoroughly protects the structural integrity and proper function of the intestinal barrier across multiple dimensions, including homeostasis maintenance, injury repair, and toxin defense (summarized in **Figure 3**).

### 4.4 Maintainers of immune homeostasis

The interaction between lamina propria macrophages and group 3 innate lymphoid cells (ILC3s) plays a pivotal role in fine-tuning intestinal immune homeostasis. Microbial stimulation induces local CX3CR1^+^lamina propria macrophages to express IL-23 and IL-1β, which subsequently activate adjacent ILC3s to produce IL-22, thereby enhancing epithelial barrier function, facilitating antimicrobial peptide secretion, and initiating tissue regeneration (80, 81). Activated ILC3s (82), through a feedback loop, enhance granulocyte-macrophage colony-stimulating factor (GM-CSF) (83) expression, which modulates the functional status of lamina propria macrophages. This reprogramming suppresses activation of detrimental pro-fibrotic pathways in lamina propria macrophages while promoting their development into a protective phenotype characterized by substantial IL-10 production and metabolic release of retinoic acid (84). IL-10 and retinoic acid synergistically enhance the development, proliferation, and functional maintenance of Treg cells in the local microenvironment, thereby reinforcing immune tolerance (85).

Lamina propria macrophages directly contribute to oral tolerance establishment through a distinct mechanism of antigen processing and presentation. CX3CR1^+^ macrophages initially utilize the CX3CL1/CX3CR1 axis to sample luminal antigens via their cellular extensions. They subsequently transfer these antigens to adjacent migratory CD103^+^dendritic cells through CX-43-dependent gap junctions (86). The dendritic cells then migrate to mesenteric lymph nodes to induce antigen-specific Treg cell generation. In this process, macrophage-derived IL-10 produced in response to microbial stimulation is essential for expansion and maintenance of Treg cells activated by specific dietary or commensal bacterial antigens, its absence specifically impairs oral tolerance (87). Moreover, unlike dendritic cells, macrophages can directly function as antigen-presenting cells by engaging with Treg progenitor cells through MHC II (88). Absence of MHC II on macrophages impedes development of antigen-specific Treg cells.

In addition to regulating Treg cells, macrophages may facilitate development or maintenance of protective commensal-specific Th17 cells by secreting IL-1βduring intestinal homeostasis (89). These cells release IL-17 and IL-22, which can enhance epithelial barrier function and antimicrobial defense (90). Through interactions with ILC3s, antigen presentation, and modulation of Treg and Th17 cell differentiation and function, lamina propria macrophages orchestrate host defense, tissue repair, and immune tolerance (summarized in **Figure 3**).

## 5 Targeting macrophages: translational frontiers from mechanism to therapy

New treatment approaches have been made possible by our expanding knowledge of the regulatory networks governing intestinal macrophages. Even if the majority of therapies now on the market indirectly alter macrophage function through nutritional, microbial, or neurological mechanisms, they nevertheless present viable options for reestablishing intestinal homeostasis. The strategies that indirectly affect macrophage phenotype and function through neuromodulation and microbiota/dietary interventions will be covered in the next sections, along with methods that focus on certain receptors or signaling cascades.

### 5.1 Receptor-specific drugs and pathway modulators

Targeting specific receptors or signaling pathways in macrophages to precisely modulate their function has emerged as a novel strategy for treating disorders of gut-brain interaction. Aryl hydrocarbon receptor agonists can mimic the actions of beneficial metabolites, directing macrophages toward an anti-inflammatory, reparative state (91). Preclinical results validate that this approach can reduce visceral hypersensitivity and mucosal inflammation. Similarly, given the pathway by which secondary bile acids inhibit macrophage inflammation via TGR5, development of TGR5-selective agonists represents a rational strategy for alleviating intestinal inflammation (92). The CX3CR1/CX3CL1 chemokine axis is a promising target for modulating macrophage recruitment and functional maintenance, its agonists may help preserve protective macrophage subsets in inflammatory contexts (93). Moreover, targeted activation of α7nAChR has been shown to ameliorate intestinal inflammation and reduce pro-inflammatory cytokine production in a murine model of postoperative ileus.

Targeting and depleting inflammatory macrophages represents a promising therapeutic strategy. In spontaneous diabetic models, Csf1_op/op_ mice, which lack macrophages, do not exhibit delayed gastric emptying (68). In contrast, Ccr2^-^/^-^ mice, which have impaired macrophage migration, display persistent muscle dysfunction. These seemingly contradictory results may reflect that the diabetic mouse model already contains M1-polarized macrophages; therefore, CSF1 inhibitors could alleviate pre-existing gastrointestinal motility abnormalities rather than prevent their development.

Neuro-immune connections offer numerous intervention opportunities. Intestinal 5-HT can modulate innate immune activity through receptors on immune cells (94), neuropeptide Y and its analogs, via their specific receptors, can affect gastrointestinal motility and immune function, positioning them as potential targets for regulating intestinal immune balance.

Advanced technologies such as single-cell transcriptomics can identify disease-specific aberrant pathways to guide therapeutic development. Identification of aberrantly active IL-12/JAK-STAT signaling and CCR2-mediated monocyte infiltration in the gastric muscularis of gastroparesis patients presents new opportunities for applying JAK inhibitors and CCR2 antagonists, while also paving the way for development of innovative therapies (67).

**Figure 4:**
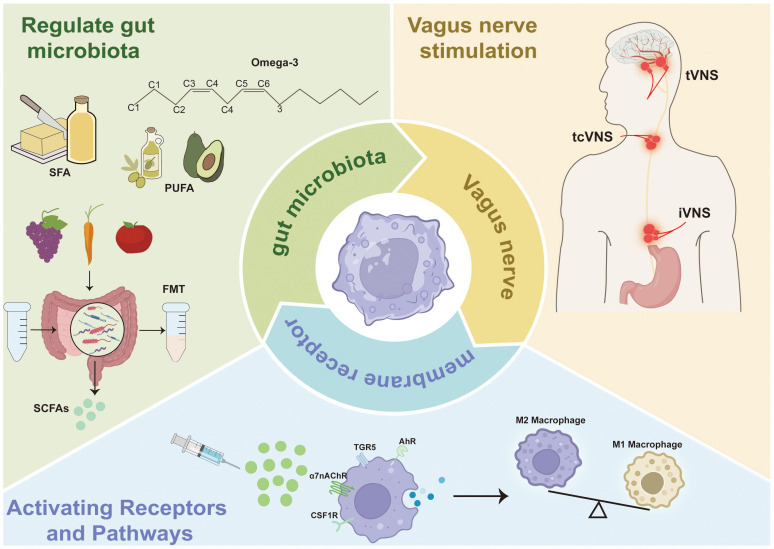
Therapeutic strategies targeting macrophages. Macrophages are an important therapeutic target for gastrointestinal motility disorders. Three complementary approaches can precisely regulate their function: molecular-targeted agents directly modulate macrophage activation; neuromodulation (e.g., vagus nerve stimulation) restores motility via anti-inflammatory pathways; and dietary or microbial interventions reshape macrophage phenotypes by altering the microenvironment. Together, these three strategies provide a complete roadmap from mechanism to clinical translation. Notably, while neuromodulatory and dietary/microbial interventions hold therapeutic promise, they influence macrophages indirectly and should be viewed as complementary to direct macrophage-targeting strategies.

In conclusion, precise modulation of macrophage function can be achieved by targeting macrophage-specific receptors or directly blocking disease-associated aberrant pathways. This offers a novel mechanism-to-clinic approach for reestablishing gut immune homeostasis and addressing related functional disorders (summarized in **Figure 4**).

### 5.2 Neuroimmune intervention

Neuroimmune regulation via vagus nerve pathways represents a vital strategy for reestablishing gastrointestinal homeostasis. Transcutaneous vagus nerve stimulation exemplifies the connection between fundamental mechanisms and therapeutic applications (95). By stimulating targeted areas such as the auricle, neck, or abdomen, it activates the body’s intrinsic physiological regulatory reflexes, producing anti-inflammatory effects and enhancing gastrointestinal motility.

This therapy has shown efficacy in multiple disorders of gut-brain interactions. In functional dyspepsia (FD) patients, four weeks of transcutaneous auricular stimulation at 10 Hz or 25 Hz resulted in considerable relief of epigastric pain in over 75% of patients, along with improvements in symptoms such as bloating and early satiety (96). In constipation-predominant irritable bowel syndrome (IBS-C) patients, it reduced abdominal pain severity by 64% and improved rectal sensation and stool passage (97). Moreover, transcutaneous electrical nerve field stimulation helps alleviate abdominal pain associated with IBS in adolescents (97). A randomized double-blind trial demonstrated that its analgesic effect was significantly superior to sham treatment, with benefits persisting for nearly 9 weeks. Another trial involving 50 adolescent IBS patients similarly confirmed its beneficial effect in reducing abdominal pain and improving overall well-being (98). This intervention can also accelerate gastric emptying in gastroparesis patients (99).

Its analgesic mechanism involves modulation of central pain processing, reduction of central sensitization, and indirect reduction of neural sensitivity through suppression of peripheral inflammation (100). Clinical studies indicate that this stimulation can reduce serum levels of pro-inflammatory cytokines; animal experiments further demonstrate that it enhances gastric motility and reduces visceral hypersensitivity while inhibiting hyperactivation of the HPA axis, suggesting that its effects may be mediated through vagovagal pathways (101). In a mouse model of constipation-predominant IBS, transcutaneous auricular stimulation improved stool consistency, facilitated transit, and reduced visceral hyperalgesia by restoring gut microbiota and increasing the interstitial cell of Cajal population (102).

The underlying mechanism involves activation of the cholinergic anti-inflammatory pathway. Electrical vagus nerve stimulation and receptor agonists can reduce intestinal inflammation and enhance motility. This effect is preserved in animals with splenic denervation or T-cell deficiency but is absent in chimeric mice with macrophage-specific deletion of α7nAChR (summarized in **Figure 4**). However, rather than directly pharmacologically targeting macrophages, vagus nerve stimulation affects them indirectly.

### 5.3 Microbiota and dietary interventions

The gut microbiota, a critical environmental determinant of macrophage phenotype and function, represents a significant intervention target for indirectly regulating macrophages and improving gastrointestinal motility disorders. Key strategies include modulating the microbial ecosystem and altering dietary composition.

Regarding microbial ecosystem modulation, direct replenishment or promotion of beneficial bacteria is essential. Certain probiotics (e.g., *Lactobacillus plantarum 299v (*103), *Lactobacillus casei GG (*104)) can foster an environment conducive to macrophage homeostasis by enhancing the mucus barrier and suppressing pathogens, among other mechanisms. Clinical studies indicate that *Bifidobacterium infantis 35624* can ameliorate IBS symptoms, with its efficacy associated with modification of immune markers including IL-10 (105). Prebiotics such as pectin can enhance short-chain fatty acid (SCFA) production, thereby steering macrophages toward an anti-inflammatory and reparative phenotype through epigenetic and other mechanisms (106). Fecal microbiota transplantation (FMT) shows promise for altering the microbiota and alleviating symptoms, although considerable variability in clinical outcomes has been observed, its long-term efficacy and mechanisms warrant further validation (107).

Dietary components can directly influence the macrophage microenvironment (108, 109). Long-chain saturated fatty acids can induce macrophage polarization toward a pro-inflammatory phenotype by activating receptors such as TLR4 and CD36, potentially contributing to the pathogenesis of disorders of gut-brain interaction (DGBI) (110, 111). Conversely, increasing ω-3 polyunsaturated fatty acid intake (112) can enhance macrophage phagocytic (113) and pro-resolving (114) capacity. Monounsaturated fatty acids such as oleic acid can reduce inflammation by inhibiting TLR4 (115) and activating PPAR-γ (116). In conclusion, probiotics, prebiotics, fecal microbiota transplantation, and optimization of dietary fatty acid and fiber intake can remodel the microbiota and its metabolite profile at multiple levels, thereby indirectly modulating macrophage function. This offers a mechanism-based, multifaceted intervention strategy for managing disorders of gut-brain interaction by addressing environmental factors (summarized in **Figure 4**).

## 6 Challenges and future perspectives

While targeting macrophages offers promising prospects for treating gastrointestinal motility disorders, the transition from molecular discovery to clinical application encounters substantial barriers, impeded by several practical obstacles.

The fundamental challenge resides in the translational divide between basic research and clinical application. The multi-system networks in the human body are highly variable and dynamic; pathway effects observed in simplified models are often obscured in complex physiological contexts. The efficacy of numerous therapies fluctuates due to the absence of patient stratification criteria. Fecal microbiota transplantation (FMT) can indirectly influence macrophages by altering the microbiota and shows potential in treating irritable bowel syndrome (117), however, its therapeutic success is markedly variable. Optimal donor selection, transplantation protocols, and long-term safety measures remain unstandardized. Intervention strategies themselves also lack standardized, mechanism-based parameters. Using transcutaneous vagus nerve stimulation as an example, stimulation parameters employed across studies vary considerably, and a standardized therapeutic framework has yet to be established (118). Moreover, interventions such as prebiotics, while theoretically capable of influencing immunity through SCFAs, frequently yield inconsistent clinical outcomes.

To overcome these challenges, it is imperative to develop systems that can recapitulate physiological complexity and enable precision intervention. Advanced three-dimensional gut organoid systems, which allow independent manipulation of luminal and tissue environments, have revealed, on millisecond-to-hour timescales, how commensal bacteria directly influence T cell fate by modulating neuronal substance P (SP) release (119). This offers a powerful platform for examining macrophage function within the intestinal network more comprehensively and accurately in an *ex vivo* context. Optogenetics and chemogenetics provide millisecond-level temporal control of specific neuronal subpopulations, enabling causal validation of their effects on macrophage polarization, microbiota modulation, and intestinal motility *in vivo*.

## 7 Conclusion

In conclusion, gastrointestinal function depends on the intricate equilibrium of a tripartite network consisting of neural, immune, and microbial elements. Macrophages, as central signaling hubs, integrate cues from neurons and microbes, and their plasticity allows them to play a dual role in both maintaining immune homeostasis and driving motility disorders. Translating these insights into clinical therapies is challenging due to network buffering effects, lack of patient stratification, and ambiguous intervention parameters. Innovation lies in developing research platforms that can simulate physiological complexity and enable precision intervention. Technologies such as three-dimensional organoids and optogenetics/chemogenetics facilitate examination of macrophage functions with high spatiotemporal resolution in human-relevant models. These approaches have the potential to elucidate the specific circuits governing gastrointestinal motility and provide transformative support for target identification, patient stratification, and personalized treatment.

## Glossary

ENS: enteric nervous system
LpM: lamina propria macrophages
MMs: muscularis macrophages
ICC: interstitial cells of Cajal
ILC3s: group 3 innate lymphoid cells
CX3CR1: C-X3-C motif chemokine receptor 1
IL-10R: interleukin-10 receptor
TNF-α: tumor necrosis factor-alpha
IL-1β: interleukin-1beta
IL-6: interleukin-6
IL-10: interleukin-10
CCL2: C-C motif chemokine ligand 2
CSF-1: colony-stimulating factor 1
GM-CSF: granulocyte-macrophage colony-stimulating factor
TGF-β: transforming growth factor-beta
MHC-II: major histocompatibility complex class II
TLR4: Toll-like receptor 4
TRAF6: TNF receptor-associated factor 6
IRAK1: interleukin-1 receptor-associated kinase 1
JAK-STAT: Janus kinase/signal transducer and activator of transcription
Jak2: Janus kinase 2
STAT3: signal transducer and activator of transcription 3
SOCS1: suppressor of cytokine signaling 1
SHIP1: SH2-containing inositol 5’-phosphatase 1
PPAR-γ: peroxisome proliferator-activated receptor gamma
AhR: aryl hydrocarbon receptor
TGR5: Takeda G protein-coupled receptor 5
GPR43: G protein-coupled receptor 43
α7nAChR: alpha-7 nicotinic acetylcholine receptor
β2AR: beta-2 adrenergic receptor
TRPV1: transient receptor potential vanilloid 1
TRPA1: transient receptor potential ankyrin 1
ACh: acetylcholine
NE: norepinephrine
5-HT: 5-hydroxytryptamine
VIP: vasoactive intestinal peptide
NPY: neuropeptide Y
CGRP: calcitonin gene-related peptide
PGE2: prostaglandin E2
NO: nitric oxide
SCFAs: short-chain fatty acids
Arg1: arginase 1
iNOS: inducible nitric oxide synthase
HO-1: heme oxygenase-1
VEGF-C: vascular endothelial growth factor C
HGF: hepatocyte growth factor
BMP2: bone morphogenetic protein 2
CD163: cluster of differentiation 163
VE-cadherin: vascular endothelial cadherin
LPS: lipopolysaccharide
FD: functional dyspepsia
IBS-C: constipation-predominant irritable bowel syndrome
HPA: axis hypothalamic-pituitary-adrenal axis
